# *N*-(2,3-Dimethyl­phen­yl)-4-methylbenzamide

**DOI:** 10.1107/S1600536811034490

**Published:** 2011-08-27

**Authors:** Vinola Z. Rodrigues, Peter Herich, B. Thimme Gowda, Jozef Kožíšek

**Affiliations:** a Department of Chemistry, Mangalore University, Mangalagangotri 574 199, Mangalore, India; b Institute of Physical Chemistry and Chemical Physics, Slovak University of Technology, Radlinského 9, SK-812 37 Bratislava, Slovak Republic

## Abstract

In the mol­ecule of the title compound, C_16_H_17_NO, the two aromatic rings are almost perpendicular to each other [dihedral angle 85.90 (5)°]. The crystal structure is stabilized by inter­molecular N—H⋯O hydrogen bonds which link the mol­ecules, forming *C*(4) chains running along the *c* axis.

## Related literature

For preparation of the title compound, see: Gowda *et al.* (2003 ▶). For the study of the effect of substituents on the structures and other aspects of *N*-(ar­yl)amides, see: Arjunan *et al.* (2004 ▶); Bhat & Gowda (2000 ▶); Bowes *et al.* (2003 ▶); Gowda *et al.* (2009 ▶); Rodrigues *et al.* (2011 ▶); Saeed *et al.* (2010 ▶).
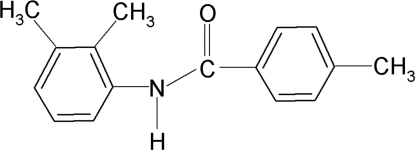


## Experimental

### 

#### Crystal data

C_16_H_17_NO
*M* *_r_* = 239.31
Monoclinic, 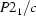

*a* = 8.1723 (3) Å
*b* = 19.3923 (7) Å
*c* = 9.3170 (3) Å
β = 111.781 (4)°
*V* = 1371.14 (9) Å^3^
*Z* = 4
Mo *K*α radiation
μ = 0.07 mm^−1^
*T* = 293 K
0.76 × 0.12 × 0.09 mm

#### Data collection

Oxford Xcalibur Ruby Gemini diffractometer
Absorption correction: analytical [*CrysAlis RED* (Oxford Diffraction, 2009 ▶) based on Clark & Reid (1995 ▶)] *T*_min_ = 0.989, *T*_max_ = 0.994
21529 measured reflections
3806 independent reflections
1925 reflections with *I* > 2σ(*I*)
*R*_int_ = 0.030

#### Refinement

*R*[*F* ^2^ > 2σ(*F* ^2^)] = 0.042
*wR*(*F* ^2^) = 0.126
*S* = 0.93
3806 reflections
166 parameters
H-atom parameters constrained
Δρ_max_ = 0.20 e Å^−3^
Δρ_min_ = −0.18 e Å^−3^

### 

Data collection: *CrysAlis CCD* (Oxford Diffraction, 2009 ▶); cell refinement: *CrysAlis CCD*; data reduction: *CrysAlis RED* (Oxford Diffraction, 2009 ▶); program(s) used to solve structure: *SHELXS97* (Sheldrick, 2008 ▶); program(s) used to refine structure: *SHELXL97* (Sheldrick, 2008 ▶); molecular graphics: *DIAMOND* (Brandenburg, 2002 ▶); software used to prepare material for publication: *enCIFer* (Allen *et al.*, 2004 ▶).

## Supplementary Material

Crystal structure: contains datablock(s) I, global. DOI: 10.1107/S1600536811034490/bt5622sup1.cif

Structure factors: contains datablock(s) I. DOI: 10.1107/S1600536811034490/bt5622Isup2.hkl

Supplementary material file. DOI: 10.1107/S1600536811034490/bt5622Isup3.cml

Additional supplementary materials:  crystallographic information; 3D view; checkCIF report

## Figures and Tables

**Table 1:** Hydrogen-bond geometry (Å, °)

*D*—H⋯*A*	*D*—H	H⋯*A*	*D*⋯*A*	*D*—H⋯*A*
N1—H1*A*⋯O1^i^	0.86	2.20	2.9256 (12)	143

Symmetry code: (i) 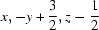.

**Fig. 1.**
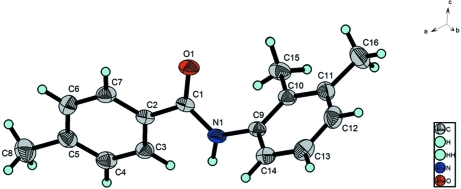
Molecular structure of the title compound showing the atom labelling scheme. Displacement ellipsoids are drawn at the 50% probability level. H atoms are represented as small spheres of arbitrary radii.

**Fig. 2.**
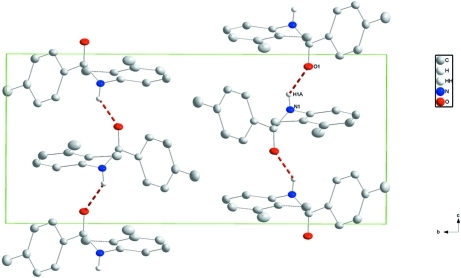
Part of the crystal structure of the title compound generated by N—H···O hydrogen bonds which are shown by dashed lines. H atoms not involved in intermolecular bonding have been omitted.

## supplementary crystallographic information

### Comment

The structural aspects of *N*-aryl amides are of interest due to their chemical and biological importance (Arjunan *et al.*, 2004; Bhat & Gowda, 2000; Bowes *et al.*, 2003; Gowda *et al.*, 2003; Saeed *et al.*, 2010). In the present work, as part of a study of the substituent effects on the structures of benzanilides (Gowda *et al.*, 2003, 2009; Rodrigues *et al.*, 2011), the structure of 4-methyl-*N*-(2,3-dimethylphenyl)benzamide (I) has been determined (Fig. 1). In the crystal, the *ortho*- and *meta*-methyl substituents in the anilino ring are positioned *anti* to the N—H bond, similar to that observed in one of the molecules of 4-methyl-*N*-(2-methylphenyl)benzamide (II) (Rodrigues *et al.*, 2011).

The central amide group –NHCO– is tilted to the anilino ring with the C10—C9—N1—C1 and C14—C9—N1—C1 torsion angles of -63.4 (2)° and 118.1 (1)°. The C3—C2—C1—N1 and C7—C2—C1—N1 torsion angles are -24.4 (2)° and 156.8 (1)°, respectively, while the C3—C2—C1—O1 and C7—C2—C1—O1 torsion angles are 155.6 (1)° and -23.2 (2)°, respectively. But the C2—C1—N1—C9 and C9—N1—C1—O1 torsion angles are -179.5 (1)° and 0.5 (2)°, respectively.

The packing of molecules linked by N—H···O hydrogen bonds is shown in Fig. 2.

### Experimental

The title compound was prepared according to the method described by Gowda *et al.* (2003). The purity of the compound was checked by determining its melting point. It was characterized by recording its infrared and NMR spectra. Cuboid-like colourless single crystals of the title compound were obtained by slow evaporation from an ethanol solution of the compound (0.5 g in about 30 ml of ethanol) at room temperature.

### Refinement

All H atoms were visible in difference maps and then treated as riding atoms with C—H distances of 0.93Å (C-aromatic), 0.96Å (C-methyl) and N—H = 0.86 Å. The *U*_iso_(H) values were set at 1.2*U*_eq_(C-aromatic, N) and 1.5*U*_eq_(*C*-methyl).

### Crystal data

**Table:**

C_16_H_17_NO	*F*(000) = 512
*M**_r_* = 239.31	*D*_x_ = 1.159 Mg m^−^^3^
Monoclinic, *P*2_1_/*c*	Mo *K*α radiation, λ = 0.71073 Å
Hall symbol: -P 2ybc	Cell parameters from 7558 reflections
*a* = 8.1723 (3) Å	θ = 3.5–29.5°
*b* = 19.3923 (7) Å	µ = 0.07 mm^−^^1^
*c* = 9.3170 (3) Å	*T* = 293 K
β = 111.781 (4)°	Cuboid, colourless
*V* = 1371.14 (9) Å^3^	0.76 × 0.12 × 0.09 mm
*Z* = 4	

### Data collection

**Table:**

Oxford Xcalibur Ruby Gemini diffractometer	3806 independent reflections
Radiation source: fine-focus sealed tube	1925 reflections with *I* > 2σ(*I*)
graphite	*R*_int_ = 0.030
Detector resolution: 10.4340 pixels mm^-1^	θ_max_ = 29.5°, θ_min_ = 3.5°
ω scans	*h* = −10→11
Absorption correction: analytical [*CrysAlis RED* (Oxford Diffraction, 2009). Analytical numeric absorption correction using a multifaceted crystal model based on expressions derived by Clark & Reid (1995).]	*k* = −24→26
*T*_min_ = 0.989, *T*_max_ = 0.994	*l* = −12→12
21529 measured reflections	

### Refinement

**Table:**

Refinement on *F*^2^	Primary atom site location: structure-invariant direct methods
Least-squares matrix: full	Secondary atom site location: difference Fourier map
*R*[*F*^2^ > 2σ(*F*^2^)] = 0.042	Hydrogen site location: inferred from neighbouring sites
*wR*(*F*^2^) = 0.126	H-atom parameters constrained
*S* = 0.93	*w* = 1/[σ^2^(*F*_o_^2^) + (0.0738*P*)^2^] where *P* = (*F*_o_^2^ + 2*F*_c_^2^)/3
3806 reflections	(Δ/σ)_max_ = 0.001
166 parameters	Δρ_max_ = 0.20 e Å^−^^3^
0 restraints	Δρ_min_ = −0.18 e Å^−^^3^

### Special details

**Table:**

Geometry. All e.s.d.'s (except the e.s.d. in the dihedral angle between two l.s. planes) are estimated using the full covariance matrix. The cell e.s.d.'s are taken into account individually in the estimation of e.s.d.'s in distances, angles and torsion angles; correlations between e.s.d.'s in cell parameters are only used when they are defined by crystal symmetry. An approximate (isotropic) treatment of cell e.s.d.'s is used for estimating e.s.d.'s involving l.s. planes.
Refinement. Refinement of *F*^2^ against ALL reflections. The weighted *R*-factor *wR* and goodness of fit *S* are based on *F*^2^, conventional *R*-factors *R* are based on *F*, with *F* set to zero for negative *F*^2^. The threshold expression of *F*^2^ > σ(*F*^2^) is used only for calculating *R*-factors(gt) *etc*. and is not relevant to the choice of reflections for refinement. *R*-factors based on *F*^2^ are statistically about twice as large as those based on *F*, and *R*-factors based on ALL data will be even larger.

### Fractional atomic coordinates and isotropic or equivalent isotropic displacement parameters (Å^2^)

**Table:**

	*x*	*y*	*z*	*U*_iso_*/*U*_eq_	
C1	0.73885 (17)	0.70346 (6)	0.43887 (13)	0.0447 (3)	
C2	0.84624 (17)	0.65196 (6)	0.39419 (13)	0.0440 (3)	
C3	0.80527 (19)	0.62986 (7)	0.24275 (14)	0.0512 (3)	
H3A	0.7064	0.6474	0.1642	0.061*	
C4	0.9099 (2)	0.58240 (7)	0.20867 (15)	0.0572 (4)	
H4A	0.8805	0.5684	0.1067	0.069*	
C5	1.0570 (2)	0.55487 (7)	0.32099 (16)	0.0571 (4)	
C6	1.0959 (2)	0.57579 (8)	0.47215 (17)	0.0613 (4)	
H6A	1.1935	0.5573	0.5504	0.074*	
C7	0.9927 (2)	0.62340 (7)	0.50858 (14)	0.0552 (4)	
H7A	1.0213	0.6366	0.6110	0.066*	
C8	1.1734 (3)	0.50369 (9)	0.2821 (2)	0.0863 (5)	
H8C	1.2149	0.4698	0.3627	0.104*	
H8B	1.2721	0.5274	0.2727	0.104*	
H8A	1.1072	0.4813	0.1861	0.104*	
C9	0.53294 (17)	0.79984 (6)	0.35140 (12)	0.0433 (3)	
C10	0.38966 (17)	0.78287 (7)	0.39137 (13)	0.0476 (3)	
C11	0.29061 (19)	0.83671 (9)	0.41903 (14)	0.0579 (4)	
C12	0.3352 (2)	0.90411 (9)	0.40209 (17)	0.0671 (4)	
H12A	0.2703	0.9397	0.4221	0.081*	
C13	0.4729 (2)	0.92024 (8)	0.35643 (15)	0.0635 (4)	
H13A	0.4983	0.9660	0.3431	0.076*	
C14	0.57237 (19)	0.86776 (7)	0.33078 (14)	0.0519 (3)	
H14A	0.6655	0.8779	0.2998	0.062*	
C15	0.3376 (2)	0.70931 (8)	0.39997 (17)	0.0644 (4)	
H15C	0.2119	0.7050	0.3519	0.077*	
H15B	0.3751	0.6955	0.5063	0.077*	
H15A	0.3926	0.6804	0.3473	0.077*	
C16	0.1334 (2)	0.82157 (11)	0.4618 (2)	0.0875 (6)	
H16C	0.0944	0.8634	0.4942	0.105*	
H16B	0.1659	0.7887	0.5448	0.105*	
H16A	0.0398	0.8030	0.3738	0.105*	
N1	0.64148 (14)	0.74732 (5)	0.32655 (10)	0.0464 (3)	
H1A	0.6453	0.7433	0.2359	0.056*	
O1	0.74010 (13)	0.70534 (5)	0.57153 (9)	0.0590 (3)	

### Atomic displacement parameters (Å^2^)

**Table:**

	*U*^11^	*U*^22^	*U*^33^	*U*^12^	*U*^13^	*U*^23^
C1	0.0527 (8)	0.0490 (7)	0.0389 (6)	−0.0046 (6)	0.0246 (6)	−0.0015 (5)
C2	0.0533 (8)	0.0454 (7)	0.0388 (6)	−0.0039 (6)	0.0235 (6)	0.0006 (5)
C3	0.0606 (9)	0.0540 (8)	0.0424 (7)	0.0058 (7)	0.0229 (6)	−0.0014 (6)
C4	0.0755 (10)	0.0551 (8)	0.0476 (7)	0.0004 (7)	0.0306 (7)	−0.0053 (6)
C5	0.0686 (10)	0.0479 (8)	0.0666 (9)	0.0022 (7)	0.0387 (8)	0.0003 (6)
C6	0.0609 (9)	0.0601 (9)	0.0614 (8)	0.0103 (7)	0.0211 (7)	0.0072 (7)
C7	0.0664 (9)	0.0594 (9)	0.0411 (7)	0.0012 (7)	0.0215 (6)	0.0004 (6)
C8	0.0961 (13)	0.0766 (12)	0.1019 (13)	0.0218 (10)	0.0550 (11)	0.0016 (9)
C9	0.0481 (8)	0.0494 (8)	0.0337 (6)	−0.0015 (6)	0.0169 (5)	−0.0014 (5)
C10	0.0474 (8)	0.0599 (9)	0.0355 (6)	−0.0048 (6)	0.0154 (5)	−0.0020 (5)
C11	0.0486 (8)	0.0800 (11)	0.0440 (7)	0.0053 (8)	0.0159 (6)	−0.0056 (7)
C12	0.0661 (10)	0.0710 (11)	0.0602 (9)	0.0180 (8)	0.0186 (8)	−0.0097 (7)
C13	0.0714 (11)	0.0506 (8)	0.0600 (9)	0.0010 (8)	0.0145 (8)	−0.0013 (6)
C14	0.0550 (8)	0.0532 (8)	0.0473 (7)	−0.0053 (7)	0.0186 (6)	0.0015 (6)
C15	0.0630 (10)	0.0735 (10)	0.0602 (8)	−0.0178 (8)	0.0270 (7)	0.0017 (7)
C16	0.0647 (11)	0.1299 (16)	0.0789 (11)	0.0118 (11)	0.0395 (9)	−0.0020 (10)
N1	0.0596 (7)	0.0515 (6)	0.0367 (5)	0.0031 (5)	0.0277 (5)	0.0012 (4)
O1	0.0775 (7)	0.0704 (7)	0.0385 (5)	0.0099 (5)	0.0324 (5)	0.0019 (4)

### Geometric parameters (Å, °)

**Table:**

C1—O1	1.2327 (13)	C9—C10	1.3935 (18)
C1—N1	1.3542 (16)	C9—N1	1.4248 (16)
C1—C2	1.4873 (17)	C10—C11	1.402 (2)
C2—C7	1.3885 (19)	C10—C15	1.4991 (19)
C2—C3	1.3913 (17)	C11—C12	1.381 (2)
C3—C4	1.3711 (19)	C11—C16	1.509 (2)
C3—H3A	0.9300	C12—C13	1.379 (2)
C4—C5	1.376 (2)	C12—H12A	0.9300
C4—H4A	0.9300	C13—C14	1.378 (2)
C5—C6	1.385 (2)	C13—H13A	0.9300
C5—C8	1.509 (2)	C14—H14A	0.9300
C6—C7	1.375 (2)	C15—H15C	0.9600
C6—H6A	0.9300	C15—H15B	0.9600
C7—H7A	0.9300	C15—H15A	0.9600
C8—H8C	0.9600	C16—H16C	0.9600
C8—H8B	0.9600	C16—H16B	0.9600
C8—H8A	0.9600	C16—H16A	0.9600
C9—C14	1.3861 (18)	N1—H1A	0.8600
			
O1—C1—N1	122.69 (12)	C9—C10—C11	118.22 (12)
O1—C1—C2	120.95 (11)	C9—C10—C15	121.48 (13)
N1—C1—C2	116.36 (10)	C11—C10—C15	120.27 (13)
C7—C2—C3	118.04 (12)	C12—C11—C10	119.27 (14)
C7—C2—C1	118.87 (10)	C12—C11—C16	120.03 (15)
C3—C2—C1	123.07 (12)	C10—C11—C16	120.67 (15)
C4—C3—C2	120.36 (13)	C13—C12—C11	121.99 (14)
C4—C3—H3A	119.8	C13—C12—H12A	119.0
C2—C3—H3A	119.8	C11—C12—H12A	119.0
C3—C4—C5	121.90 (12)	C14—C13—C12	119.20 (14)
C3—C4—H4A	119.0	C14—C13—H13A	120.4
C5—C4—H4A	119.0	C12—C13—H13A	120.4
C4—C5—C6	117.77 (13)	C13—C14—C9	119.69 (14)
C4—C5—C8	121.57 (13)	C13—C14—H14A	120.2
C6—C5—C8	120.66 (14)	C9—C14—H14A	120.2
C7—C6—C5	121.17 (13)	C10—C15—H15C	109.5
C7—C6—H6A	119.4	C10—C15—H15B	109.5
C5—C6—H6A	119.4	H15C—C15—H15B	109.5
C6—C7—C2	120.73 (12)	C10—C15—H15A	109.5
C6—C7—H7A	119.6	H15C—C15—H15A	109.5
C2—C7—H7A	119.6	H15B—C15—H15A	109.5
C5—C8—H8C	109.5	C11—C16—H16C	109.5
C5—C8—H8B	109.5	C11—C16—H16B	109.5
H8C—C8—H8B	109.5	H16C—C16—H16B	109.5
C5—C8—H8A	109.5	C11—C16—H16A	109.5
H8C—C8—H8A	109.5	H16C—C16—H16A	109.5
H8B—C8—H8A	109.5	H16B—C16—H16A	109.5
C14—C9—C10	121.54 (12)	C1—N1—C9	123.11 (9)
C14—C9—N1	117.78 (11)	C1—N1—H1A	118.4
C10—C9—N1	120.67 (11)	C9—N1—H1A	118.4
			
O1—C1—C2—C7	−23.19 (18)	C14—C9—C10—C15	174.52 (11)
N1—C1—C2—C7	156.80 (12)	N1—C9—C10—C15	−3.88 (17)
O1—C1—C2—C3	155.62 (13)	C9—C10—C11—C12	1.61 (17)
N1—C1—C2—C3	−24.40 (18)	C15—C10—C11—C12	−176.44 (12)
C7—C2—C3—C4	−1.4 (2)	C9—C10—C11—C16	179.66 (12)
C1—C2—C3—C4	179.75 (12)	C15—C10—C11—C16	1.61 (18)
C2—C3—C4—C5	0.2 (2)	C10—C11—C12—C13	1.0 (2)
C3—C4—C5—C6	1.0 (2)	C16—C11—C12—C13	−177.06 (14)
C3—C4—C5—C8	−178.80 (14)	C11—C12—C13—C14	−1.8 (2)
C4—C5—C6—C7	−1.1 (2)	C12—C13—C14—C9	−0.10 (19)
C8—C5—C6—C7	178.74 (15)	C10—C9—C14—C13	2.77 (17)
C5—C6—C7—C2	−0.1 (2)	N1—C9—C14—C13	−178.78 (11)
C3—C2—C7—C6	1.4 (2)	O1—C1—N1—C9	0.50 (19)
C1—C2—C7—C6	−179.77 (13)	C2—C1—N1—C9	−179.49 (10)
C14—C9—C10—C11	−3.50 (17)	C14—C9—N1—C1	118.12 (13)
N1—C9—C10—C11	178.09 (10)	C10—C9—N1—C1	−63.42 (16)

### Hydrogen-bond geometry (Å, °)

**Table:**

*D*—H···*A*	*D*—H	H···*A*	*D*···*A*	*D*—H···*A*
N1—H1A···O1^i^	0.86	2.20	2.9256 (12)	143.

Symmetry codes: (i) *x*, −*y*+3/2, *z*−1/2.
